# Identification of differentially expressed miRNAs in differentiating benign from malignant pleural effusion

**DOI:** 10.1186/s41065-020-00119-z

**Published:** 2020-02-26

**Authors:** Quanlei Bao, Yaping Xu, Ming Ding, Ping Chen

**Affiliations:** Respiratory Medicine Department, Affiliated hospital of Jiangsu University, Zhenjiang, Jiangsu 212000 People’s Republic of China

**Keywords:** Pleural effusion, Tuberculosis pleural effusion, Malignant pleural effusion, miRNA, Receiver operating characteristic

## Abstract

**Background:**

Tuberculosis pleural effusion (TPE) and malignant pleural effusion (MPE) are very common clinical complications. Considering the totally different prognosis and clinical treatment of TPE and MPE, the accurate and non-invasive diagnosis are very critical for patients with pleural effusion to initiate efficient management and treatment. However, effective clinical biomarkers were rarely explored to distinguish benign from MPE. The purpose of this study is to identify potential miRNAs which can probably be used to differentiate malignant pleural effusion from TPE.

**Results:**

A total of 23 significantly differentially expressed miRNAs were identified in MPE, with 18 up-expressed and 5 down-expressed. And the target genes of the miRNAs mainly involved in the biology process of nervous system, cancer, immune system and metabolic process etc. Three high confident target genes, *AGO4*, *FGF9* and *LEF1* can be regulated by miR-195-5p, miR-182-5p and miR-34a-5p respectively. And these genes participate in the canonical pathway of regulation of the Epithelial-Mesenchymal and the biological functions of apoptosis, growth of tumor and cell proliferation of tumor cell lines. Further, RT-PCR validation results based on 64 collected individuals showed that the expression levels of the three miRNAs were 2–5 times higher in MPE samples, which were consistent with the microarray results. In addition, ROC curve analysis demonstrated that the combination of the three miRNAs can achieve higher AUC of 0.93 (*p*-value< 0.0001) to differentiate MPE from TPE.

**Conclusions:**

The identified miR-195-5p, miR-182-5p and miR-34a-5p can become potential diagnostic biomarkers for MPE with further evidences.

## Background

Various causes can lead to pleural effusion, and one type of effusion caused by tuberculous pleurisy was complicate and time-consuming to differentiate from the other type caused by cancer [20]. Tuberculosis caused Tuberculosis pleural effusion (TPE) is endemic especially in the developing countries [35], whereas malignant disease like lung cancer is another major factor of pleural effusion, namely malignant pleural effusion (MPE) [17, 25].

The traditional methods to diagnose TPE are based on the microbiological analyses of *M.tuberculosis* in pleural fluid, sputum and pleural biopsy specimens [25]. MPE is characterized by the manifestation of neoplastic cells in the pleural region, and the golden standard diagnostic approach is cytology, which can be used to confirm 30–60% MPE in advanced disease stages [29]. In addition, various biomarkers including vascular endothelial growth factor (*VEGF*), tumor necrosis factor (*TNF*), endothelin, interleukin (*IL*)-27, *IFN-γ* and *IL-6* have also been used in the distinction analysis of TPE and MPE [14, 33]. Given the low diagnostic yield of pleural fluid cytology and the invasive nature of pleural biopsy, it has limited the clinical application of biochemical tests [2, 22].

Our primary objective of this study was to identify potential miRNA biomarkers which can probably be used to differentiate MPE from TPE, considering the wide difference in prognosis and clinical treatment between TPE and MPE and the limitation of biochemical testing. MicroRNAs (miRNAs) are a type of small non-coding RNA with good stableness, which can mediate RNA silencing and regulate gene expression. And some specific miRNAs have been found to be critical in the development of MPE in recent years [1, 9, 26, 27]. However, most of the previous studies were still mainly based on low-throughput technologies.

## Results

### Identification of differentially expressed miRNAs

**Fig. 1:**
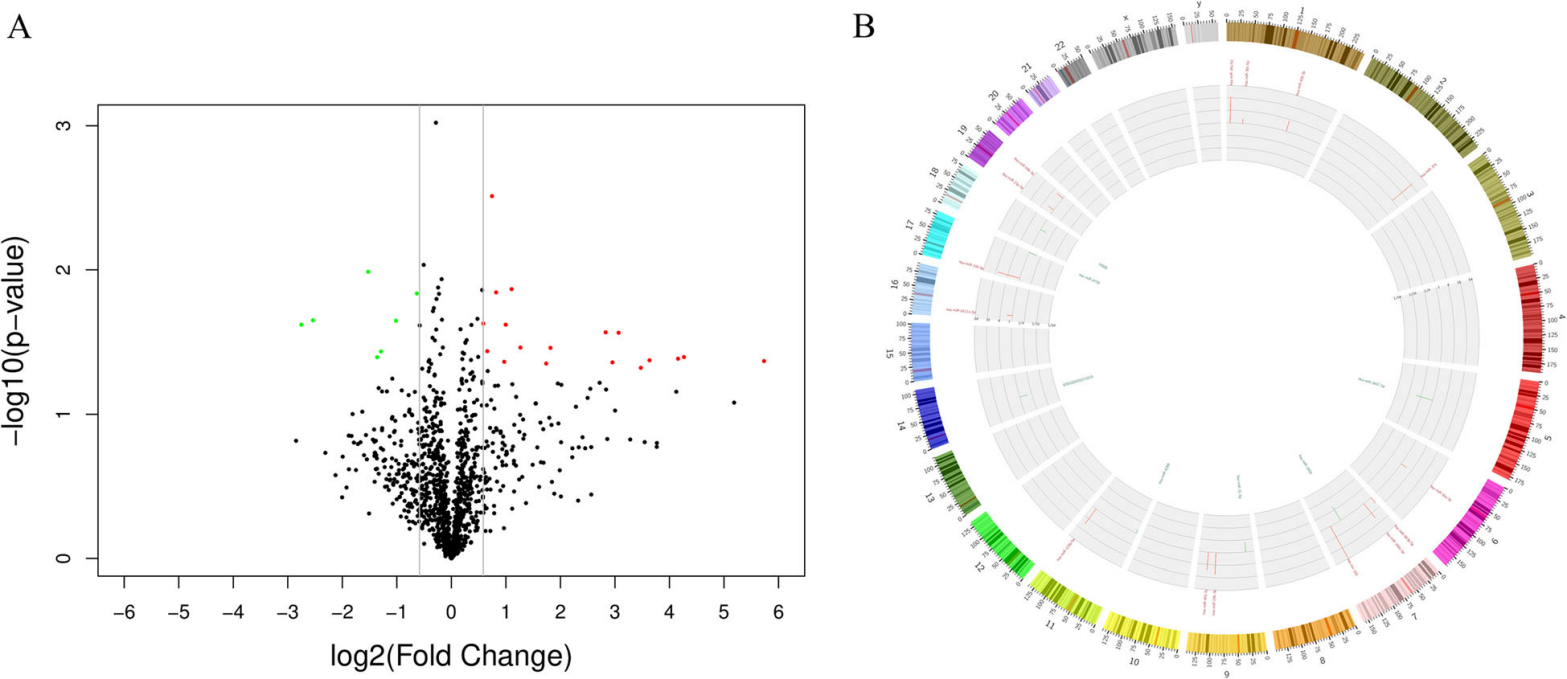
The identified differentially expressed miRNAs. **a** Volcano plot of differentially expressed miRNAs in malignant pleural effusion. **b** Chromosome locations of differentially expressed miRNAs

**Fig. 2:**
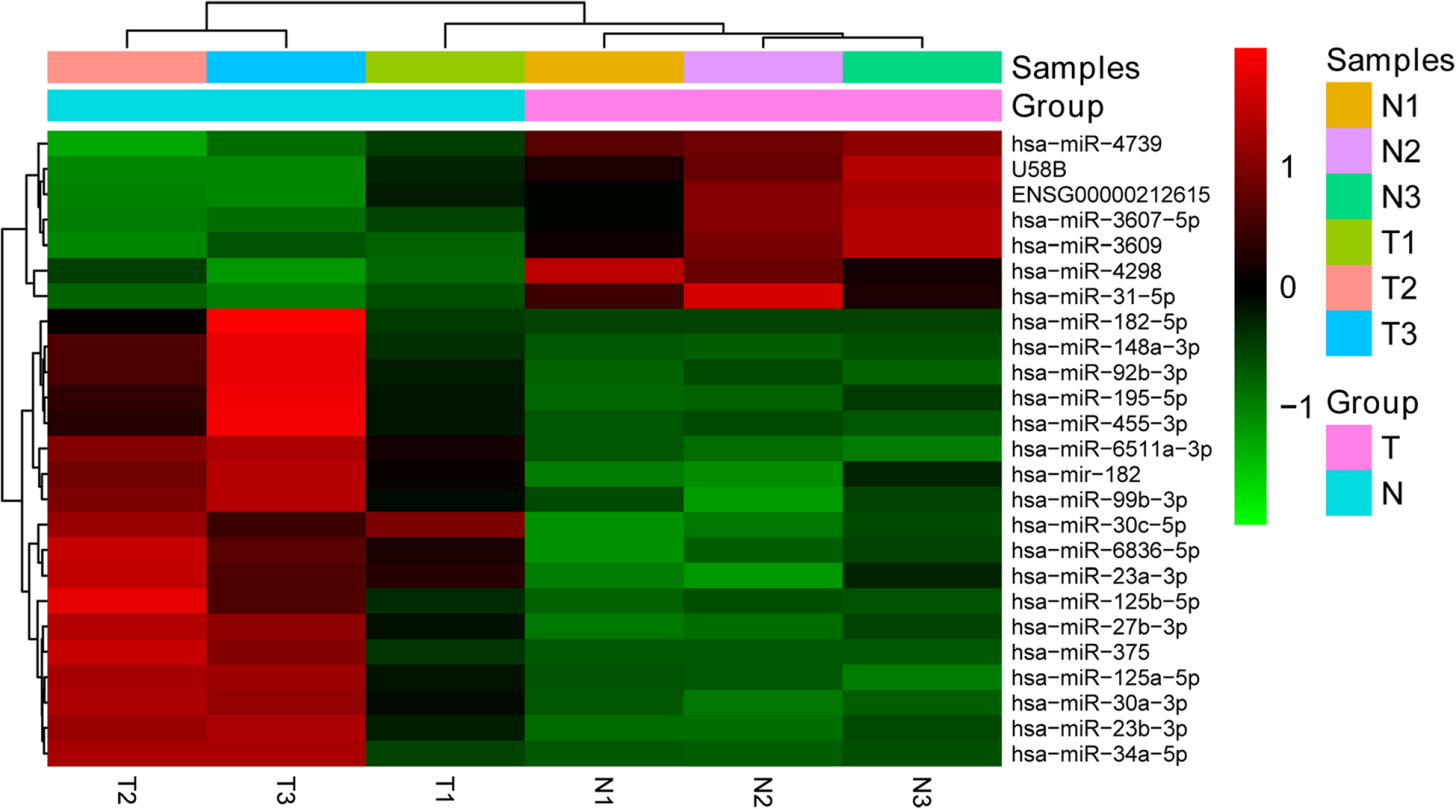
Heatmap of differentially expressed miRNAs. The group of N represents tuberculosis pleural effusion and the group of T represents malignant pleural effusion

A total of 23 miRNAs were identified significantly differentially expressed in MPE comparing with TPE after background correction and normalization. Among the miRNAs, 18 were high-expressed, and 5 were down-expressed (Fig. 1a). The chromosome locations of the miRNAs were annotated and shown in Fig. 1b. Meanwhile, based on the expression values of the differentially expressed miRNAs the enrolled samples can also be clearly clustered into two groups, one belongs to MPE and the other one was TPE (Fig. 2).

### miRNA target gene predication and functional enrichment

**Fig. 3:**
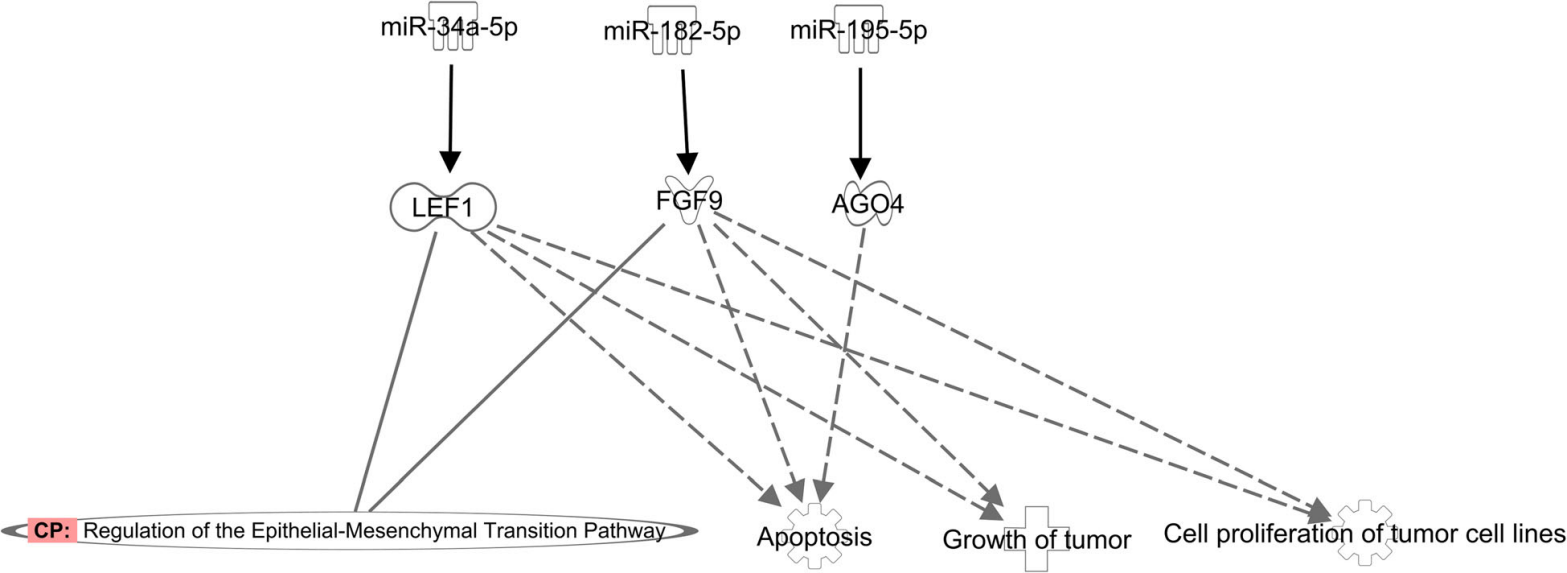
Regulation mechanism of three high confident target genes and miRNAs, and related biological functions

**Fig. 4:**
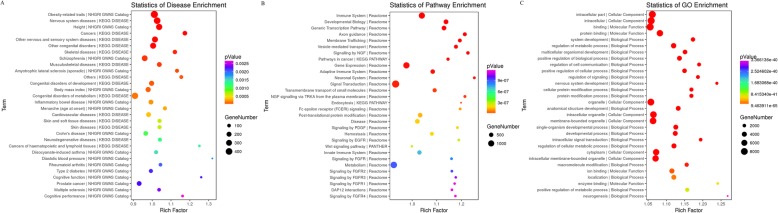
Function annotation results of differentially expressed miRNAs target genes. **a** top 30 significantly enriched diseases. **b** top 30 significantly enriched pathways. **c** top 30 significantly enriched Gene Ontology terms

miRWalk2.0 analysis showed that a total of 1356 genes can be regulated by the differentially expressed miRNAs, of which three genes, *LEF1*, *FGF9* and *AGO4*, were predicted to be high confident target genes because they were predicted to be miRNA target by all 12 tools. Also, these three genes participate in the canonical pathway of regulation of the Epithelial-Mesenchymal and the biological functions of apoptosis, growth of tumor and cell proliferation of tumor cell lines (Fig. 3). Functional annotation of all target genes using DAVID showed that the genes mainly involved in the biology process of nervous system, cancer, immune system and metabolic process etc. (Fig. 4).

### RT-PCR validation and ROC curve analysis

**Fig. 5:**
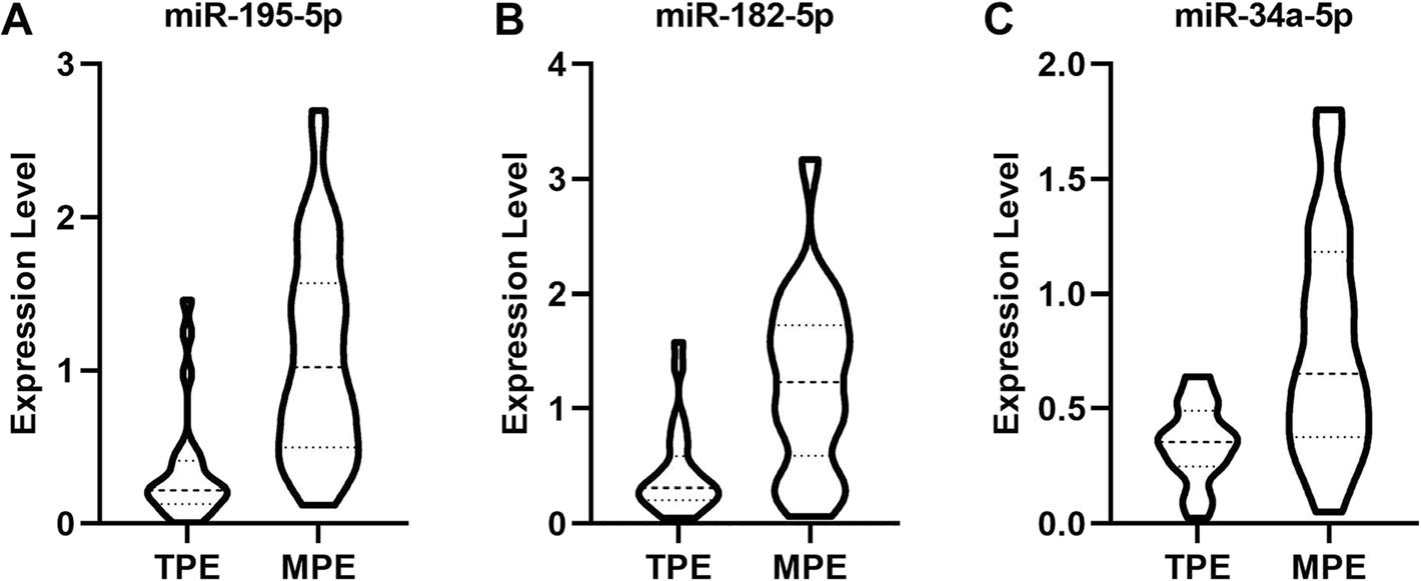
The expression levels of three key miRNAs in 65 tuberculosis pleural effusion and malignant pleural effusion individuals

Further, the expression levels of the three identified key miRNAs, miR-195-5p, miR-182-5p and miR-34a-5p, were validated using 65 collected individuals. As shown in Fig. 5, the expression level of miR-195-5p was higher in MPE group (1.10 ± 0.67) than TPE group (0.35 ± 0.30). The expression level of miR-182-5p was 1.24 ± 0.76 in MPE group and 0.45 ± 0.38 in TPE group. Similarly, higher expression level of miR-34a-5p was observed in MPE (0.79 ± 0.48) than TPE (0.35 ± 0.16). And, the differences for the three miRNAs were statistically significant between the two groups.

**Fig. 6:**
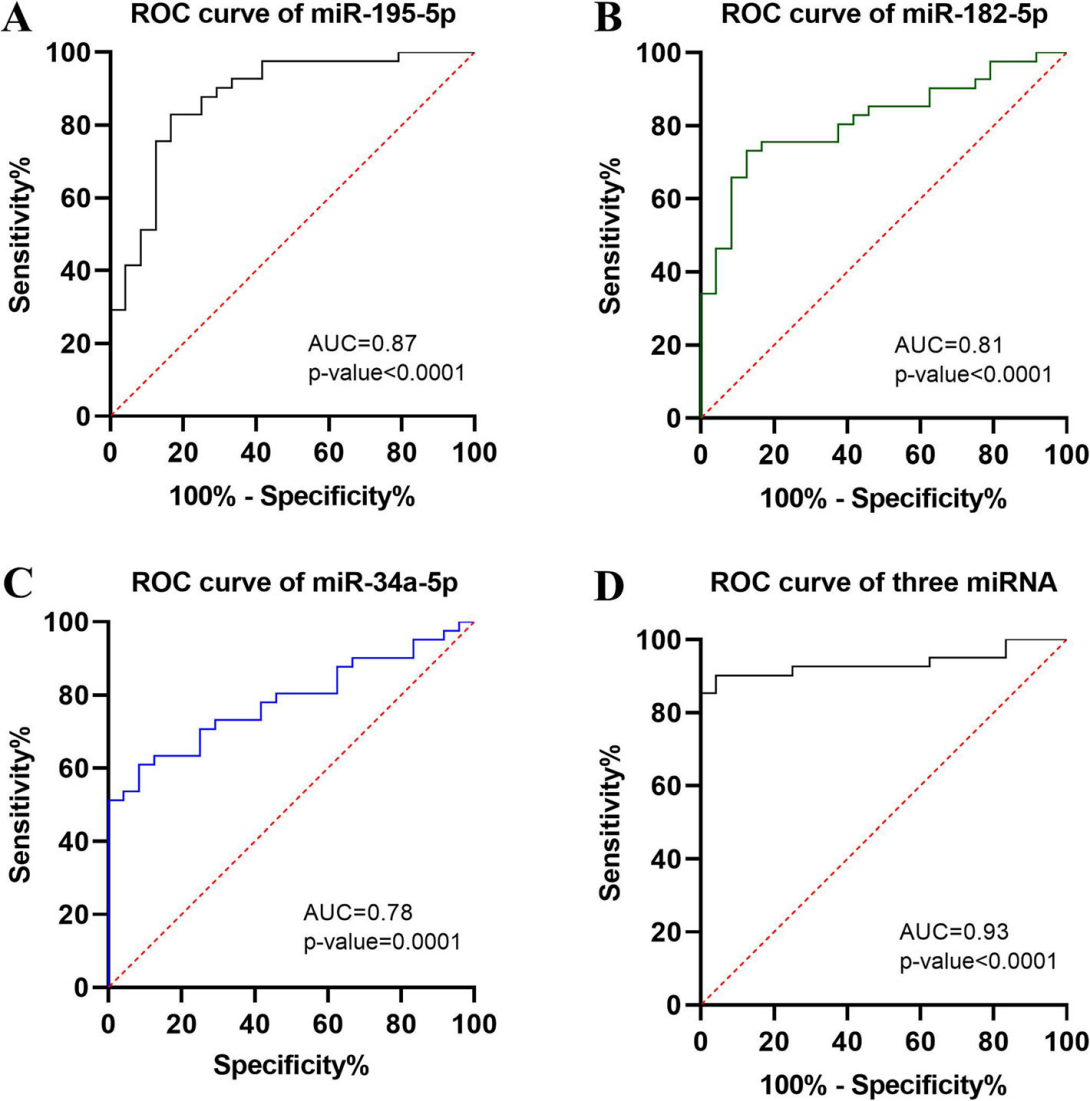
Comparison of the sensitivity and specificity of the three miRNAs and combination in differentiating tuberculosis pleural effusion from malignant pleural effusion

ROC curve analysis was further carried out to assess diagnostic ability of the three miRNAs. As shown in Fig. 6, the AUC values for miRNA-195-5p, miRNA-182-5p and miRNA-34a-5p were 0.87 (95% CI, 0.79 to 0.97), 0.81 (95% CI, 0.71 to 0.92) and 0.78 (95% CI, 0.67 to 0.89), respectively. While, the AUC for the combination of the three miRNAs can achieve 0.93 with 95% CI range from 0.87 to 0.98 (Fig. 6d).

## Discussion

In this study, a total of 23 significantly DE miRNAs were identified between MPE and TPE. Further functional annotation showed that the target genes of the differentially expressed miRNAs mainly involved in the process of cancer, immune system, regulation of metabolic process, and positive regulation of biological process, all of which are associated with benign or malignant lesions. In addition, three high confident target genes, *AGO4*, *FGF9* and *LEF1*, can be regulated by differentially expressed miR-195-5p, miR-182-5p and miR-34a-5p respectively. Also, the genes participate in the canonical pathway of regulation of the Epithelial-Mesenchymal and the biological functions of apoptosis, growth of tumor and cell proliferation of tumor cell lines. RT-PCR validation and ROC curve analysis results further demonstrated that the combination of the three differentially expressed miRNAs could become good potential non-invasive biomarkers for the diagnosis of MPE, with AUC as high as 0.93.

The expression level of miR-195-5p was significantly up-expressed in MPE than TPE. It has been demonstrated that miR-195-5p could result in a 20% decrease in *VEGF* expression via *VEGF* 3′-UTR [34] and downregulate the protein level of *HSP90B1* [5]. *VEGF* can be released by both malignant and inflammatory cells, and various studies indicated that *VEGF* is associated with survival and pleurodesis outcomes in MPE [6, 13]. Both *VEGF* and *HSP90B1* are critical in the development of MPE by regulating angiogenesis [36]. *VEGF* has also been used as drug target of Bevacizumab and Cisplatin for the treatment of MPE in advanced stage non-small-cell lung cancer from one clinical trial by Chinese PLA General Hospital (NCT01661790). In addition, Noriko Hirayama’s study showed that the dysregulation of *VEGF* could be useful for the diagnosis of malignant pleural mesothelioma [12]. Another target gene of miR-195-5p *AGO4,* as an microRNA machinery gene, was demonstrated to be upregulated in ovarian carcinogenesis and apoptosis process [15]. In addition, miR-195-5p has been widely identified as tumor suppressor in osteosarcoma, prostate cancer, and bladder cancer [4, 7, 23].

miR-182-5p which has been reported to be an oncogene in several cancers was also highly up-regulated in MPE. The dysregulation of miR-182-5p in MPE probably participated in the development of different cancers by regulating the downstream genes. In-vitro studies showed that the knock-down of miR-182-5p in prostate cancer cell can promote cell proliferation, migration and invasion by regulating the potential targets of *FOXF2*, *RECK* and *MTSS1* [10]. While in bladder cancer miR-182-5p can regulate the expression levels of *Smad4* and *RECK*, and high-expression of miR-182-5p can significantly shorten the overall survival of bladder cancer patients [11]. By regulate the expression of *FGF9*, miR-182-5p can probably induce malignant lesions by inducing Epithelial-Mesenchymal, cell proliferation and growth of tumor cells, as various studies have identified *FGF9* participate in the tumor development such as gastric cancer [28], hepatocellular carcinoma [32], NSCLC [21] and so on. All these results indicated that as an important upstream regulator, miR-182-5p participate in the biology functions related to malignant lesions development. It will significantly simplify the testing by detecting the expression of miRNA rather than tens of gene expression in the diagnosis of malignant lesions development.

Another important miRNA identified in this study was over-expressed miR-34a-5p in MPE group which showed a malignant tendency in lesions. This statement was supported by some previous publications [22–24]. In non-small cell lung cancer, high-expression of miR-34a-5p can induce cell apoptosis by targeting *MDM4*, and increase p53 and p21 protein expressions [16]. Also, p53 can indirectly regulates *Fra-1* expression via miR-34a, and eventually inhibit cell migration, invasion in colon cancer [30]. In addition, low-expression of miR-34a-5p is correlated with high probability of relapse (*p* = 0.04) and can be used as a novel prognostic marker in NSCLC patients [8]. The oncogenic transcription factor *LEF1* as the target gene of miR-34a-5p can also promote malignant lesions via Wnt signal pathway in various cancers [18, 19]. Via the canonical pathways such p53 and Wnt signal pathway, miR-34a-5p can regulate the development of malignant lesions. Each of the three miRNAs have a high accuracy, but with the miRNA panel can achieve higher AUC of 0.93 (*p*-value< 0.0001) to differentiate TPE from MPE.In summary, three potential miRNA biomarkers in differentiating TPE from MPE were identified based on miRNA microarray, RT-PCR validation and ROC curve analysis. The miRNA panel can provide an easy and non-invasive method for the diagnosis of MPE. But the results were still preliminary due to small sample size and lack of other experiments validation such as western blot.

## Methods

### Sample collection

Six patients were enrolled for miRNA microarray analysis in this study between Sep. 30, 2017 and May. 3, 2018 from the department of Respiratory Medicine, Jiangsu University Hospital. Among these patients, three were diagnosed as TPE, and the rest were diagnosed as lung adenocarcinoma classified as MPE. Detailed clinical characterslike age, gender, cytology, pleural effusion protein, pleural LDH, pleural ADA and pleural glucose antigen were listed in Table 1. The present study was approved by the Ethics Committee of Jiangsu University Hospital, and informed consent was obtained from everyone involved in this study.

**Table 1:** Detailed clinical information of involved patients

ID	Sex	Age	Diagnosis	acid-fast bacilli	Malignant tumor cell	Cell Counts 10^6/L	monocyte ratio%	LDH u/L	ADA u/L	CEA ng/ml	CA19–9 125 u/ml
1	Male	66	tuberculous pleurisy	Negative	Negative	2200	90	280	53	1.58	209.7
2	Male	21	tuberculous pleurisy	Negative	Negative	1500	70	442	68.6	1.31	575.8
3	Female	60	tuberculous pleurisy	Negative	Negative	2900	90	196	32.1	0.61	155.8
4	Male	74	lung adenocarcinoma	Negative	Positive	1700	90	334	15	414.69	> 1000
5	Female	71	lung adenocarcinoma	Negative	Positive	1700	70	207	7.1	1307.66	> 1000
6	Male	62	lung adenocarcinoma	Negative	Positive	800	40	429	7.2	> 1500	> 1000

### RNA extraction and miRNA microarray experiment

At least 50 mL of pleural fluid was collected from each patient during thoracentesis using a sterile syringe. Total RNA was extracted using the RNeasy Mini Kit (QIAGEN, Valencia, CA) according to the manufacturer’s instructions. Then RNA samples were quantified by Qubit (Thermo Fisher Scientific Inc., Wilmington, DE) and checked for integration by Agilent Bioanalyzer 2100 (Agilent Technologies Inc., Santa Clara, CA). The amplified miRNAs were hybrid to GeneChip® miRNA 4.0 array, which containing 30,424 total mature miRNA probe sets including 2578 from human and the rest from 202 other organisms. In brief, 245 ng of total RNA was labeled with Biotin using the 3DNA Array Detection FlashTag™ Biotin HSR kit (Genisphere, Hatfield, PA, U.S.) and subsequently hybridized overnight. After washing and staining processes, the GeneChip® miRNA 4.0 arrays were scanned with the Affymetrix GeneChip Scanner 3000 7G (Affymetrix, Santa Clara, CA, U.S.). Finally, the intensity was calculated and collected using AGCC software.

### Identification of differentially expressed miRNAs

To identify the differentially expressed miRNAs, we adopted our internal optimized R script by integrating affy, Limma, Biobase etc. In general, the intensity of each probe and background probes for these samples were compared using boxplot. And the scale factors were retrieved to equalize the mean intensities of these arrays. Then, GCRMA (GeneChip Robust Multi-array Analysis) which is based on RMA were used to normalize the probe intensities by incorporating non-specific binding information [31]. Also, uninformative or duplicated probes were processed to better calculate expression levels. Finally, Limma (Linear Models for Microarray Analysis) package was used to identify differentially expressed miRNAs using t-test [24]. In this study, the cutoff for differentially expressed miRNAs were set to adjusted *p*-value ≤0.05 and |log_2_ fold change| ≥ 1.

### miRNA target gene predication and function enrichment

To explore the potential biological functions of differentially expressed miRNAs, the target genes were predicted using widely adopted miRWalk2.0 software. The software integrates 12 different predication tools to improve the predication accuracy, including miRWalk, DIANA-microTv4.0, miRanda-rel2010, mirBridge, miRDB4.0, miRmap, miRNAMap, PicTar2, PITA, FNA22v2, RNAhybrid2.1 and TargetScan6.2. Here a plausible target gene was defined as at least six tools predicted to be target of differentially expressed miRNAs. Then the GO and function enrichment were carried out using online tool DAVID (Database for Annotation, Visualization and Integrated Discovery) and Ingenuity Pathway Analysis (QIAGEN, Redwood City, CA, USA). Significantly enriched diseases, pathways and GO functions were screened out with p-value less than 0.05.

### RT-PCR validation and ROC curve analysis

To validate the results, the expression levels of three key miRNAs weremeasured in 65 collected individuals using RT-PCR (Additional file 1). Among these individuals, 24 were classified as TPE and 41 were classified as MPE. Similarly, total RNA was extracted, and reverse transcribed to cDNA using cDNA synthesis kit (QIAGEN, Valencia, CA). The experiment was carried out on Bio-Rad CFX384 Real-time PCR system with primers designed using miRprimer2 software [3]. And the expression levels were normalized using internal control miR-16 and processed using 2^-*Δ*ct^ method.

To assess diagnostic ability of the three key miRNAs for differentiating between TPE and MPE, the ROC (Receiver-Operator Characteristic) and AUC (Area Under Curve) were calculated using GraphPad Prism 8 (GraphPad Software, La Jolla, CA, USA). The combinations of miRNAs as predictor was analyzed using logistic regression algorithm.

## Supplementary information

**Additional file 1.** The detailed clinical characteritics of the validation group samples.

## Data Availability

All analyzed data related to this paper are included in this paper and additional file.

## Abbreviations

AUC: Area under curve
DAVID: Database for annotation, visualization and integrated discovery
GCRMA: GeneChip robust multi-array analysis
Limma: Linear models for microarray analysis
MPE: Malignant pleural effusion
ROC: Receiver-operator characteristic
TB: Tuberculosis
TNF: Tumor necrosis factor
TPE: Tuberculosis pleural effusion
VEGF: Vascular endothelial growth factor
